# The Effect of Transcranial Direct Current Stimulation on Basketball Performance—A Scoping Review

**DOI:** 10.3390/jcm14103354

**Published:** 2025-05-12

**Authors:** James Chmiel, Rafał Buryta

**Affiliations:** Faculty of Physical Culture and Health, Institute of Physical Culture Sciences, University of Szczecin, Al. Piastów 40B blok 6, 71-065 Szczecin, Poland

**Keywords:** tDCS, transcranial direct current stimulation, basketball, sport, non-invasive brain stimulation, neurostimulation, neuromodulation, sport performance

## Abstract

**Introduction**: Basketball performance requires not only intermittent high-intensity movements—such as sprinting, jumping, and rapid directional changes—but also rapid decision-making under cognitive and psychological stress. Transcranial direct current stimulation (tDCS) has emerged as a potential modality to enhance both physical and mental performance due to its capacity to modulate cortical excitability and promote synaptic plasticity. Although the broader literature suggests that tDCS can benefit motor performance and endurance across various sports, its specific impact on basketball remains underexplored. **Methods**: This scoping review aimed to summarize current evidence on the effects of tDCS in basketball. A comprehensive literature search was conducted across databases including PubMed/Medline, Google Scholar, and Cochrane, identifying studies published between January 2008 and February 2025. Only clinical trials investigating tDCS interventions in basketball players were included. Eleven articles met the inclusion criteria and were synthesized narratively, with a focus on stimulation parameters (site, duration, intensity) and performance outcomes (shooting accuracy, dribbling, sprinting, decision-making, fatigue). **Results**: The reviewed studies indicated that tDCS—particularly when applied over the motor cortex—was associated with moderate improvements in shooting accuracy, dribbling time, repeated-sprint performance, and decision-making under fatigue. Some studies reported delayed rather than immediate benefits, suggesting that tDCS may prime neural networks for enhanced learning and retention. However, not all findings were consistent; certain interventions produced minimal or no significant effects, especially regarding subjective mental fatigue and cognitive workload. The variability in electrode placements and stimulation protocols highlights the need for methodological standardization. **Conclusions**: Current evidence partially supports the potential of tDCS to improve specific performance domains in basketball, particularly in skill acquisition, neuromuscular efficiency, and decision-making. Nevertheless, the findings are limited by small sample sizes, heterogeneous protocols, and a lack of long-term follow-up. Future research should prioritize larger, multisite studies with standardized tDCS parameters and ecologically valid outcome measures to confirm the efficacy and practical relevance of tDCS in competitive basketball settings.

## 1. Introduction

Basketball is one of the most popular sports worldwide, as indicated by the large number of prestigious leagues in which teams compete. Sports medicine actively investigates effective strategies to enhance the performance of basketball players and maximize their potential for success. Participation in any sport requires athletes to meet specific demands, which must be addressed to achieve high-level performance. These demands are commonly categorized into two broad domains: (1) physical and (2) psychological.

### 1.1. Physical Demands

Basketball is characterized by intermittent bursts of high-intensity activity interspersed with brief recovery periods [1]. During live play, athletes frequently sprint, jump and land [2], shuffle defensively, and execute rapid changes of direction, typically lasting several seconds [3]. These repeated high-intensity actions place substantial demands on the anaerobic energy systems and require well-developed power and agility [4]. One of the most distinguishing features of basketball is frequent transitions between offense and defense [5,6]. Following a quick shot or turnover, players must quickly alternate between roles, transition to a defensive stance, pursue an opponent during a fast break, or assume an entirely new offensive position [5]. These frequent transitions require exceptional speed, reactive agility, and the capacity to sustain repeated, power-driven efforts—key physical attributes at both amateur and elite levels of play [7].

Studies tracking basketball players with time-motion analysis, video-tracking systems, and wearable devices such as accelerometers have demonstrated that during a typical 40 min game (i.e., a standard FIBA match), high-level players can cover between 5 and 10 km in total distance [8,9,10,11]. While much of this distance involves low-to-moderate intensity movement (walking or jogging in half-court offense/defense), a significant portion consists of high-intensity running, sprinting, and powerful lateral movements [12]. Elite male players often transition into maximal or near-maximal speed within short intervals, demonstrating the high-intensity nature of the sport [13].

In addition to total distance, frequent changes in velocity—accelerations and decelerations—are a vital component of basketball’s physical load [14]. Research indicates that competitive play includes dozens of explosive accelerations and decelerations per quarter [15]. Such rapid transitions place substantial demands on the neuromuscular system; in turn, athletes must develop lower-body power, reactive strength, and coordination to perform abrupt speed bursts efficiently and repeatedly [16].

Basketball also requires frequent jumping—for rebounds, contested shots, layups, shot blocks, and quick directional changes [17]. Studies have shown that players perform jumping actions roughly once per minute during in-game situations, especially at the higher levels of competition [18]. This high frequency of high-intensity vertical or horizontal jumps requires high levels of maximal power output and strength-endurance in the lower limbs [19].

Due to the sport’s emphasis on short, maximal, or near-maximal efforts, anaerobic energy systems (particularly the ATP-PC and glycolytic pathways) play a central role [20]. Blood lactate levels during competitive play typically exceed 5 mmol·L^−1^ in both men’s and women’s games, indicating a heavy reliance on short-duration, high-powered efforts [21,22,23,24,25,26]. Consequently, repeated-sprint ability is critical: players must generate powerful movements, recover rapidly, and replicate these efforts consistently throughout the game.

Although competitive play is characterized by explosive movements, it is also punctuated by brief pauses—such as timeouts, fouls, free throws, and substitutions—which allow for partial phosphocreatine resynthesis and lactate clearance [7]. Nonetheless, the frequency and density of high-intensity efforts remain substantial, meaning that only well-developed anaerobic systems can meet the demands of sustained performance over the course of a full game.

While basketball is dominated by intermittent explosive efforts, it also relies heavily on aerobic energy pathways for metabolic support, particularly during prolonged or repeated possessions [7]. Players with high levels of aerobic fitness recover more quickly between high-intensity efforts, sustain higher work rates as the game progresses, and maintain performance quality in later stages (e.g., in the final quarter) [27,28]. Heart rate (HR) data frequently indicate that basketball players perform at an average intensity exceeding 85% of their maximum HR during live play, underscoring the combined reliance on both anaerobic and aerobic systems [29,30,31,32].

### 1.2. Psychological Demands

Basketball requires not only physical prowess and tactical awareness but also well-developed psychological competencies. Athletes must exhibit a high level of attentional control, especially during tasks such as shooting, where the “quiet eye period” (i.e., the final fixation on the target) can significantly enhance accuracy [33]. Given the game’s rapid and frequent transitions, players must process extensive information—visual (e.g., positions of teammates and opponents), auditory (e.g., crowd noise, coach instructions), and tactile (e.g., physical interactions with opponents)—within fractions of a second [34]. These intense cognitive demands make attentional flexibility a critical skill. A strong sense of perceived behavioral control (i.e., the belief in one’s ability to influence outcomes on the court) allows players to integrate stimuli more effectively and maintain decision-making efficiency under pressure.

Anxiety and stress—often elevated in key moments of a game—can disrupt these cognitive processes. Competitive anxiety may redirect cognitive resources toward potential negative outcomes, decreasing the capacity to execute precise motor skills or to recognize tactical opportunities [35]. Consequently, free-throw accuracy and decision-making may decline. In such situations, coping strategies become crucial: athletes who adopt engagement-focused strategies, such as positive self-talk or problem-solving approaches, tend to manage game-related stress more effectively than those who disengage or ruminate on mistakes [36].

Throughout a game or season, mental fatigue may progressively develop as players continuously alternate between offensive and defensive roles, interpret opponents’ tactics, and regulate emotional responses. This fatigue may impair shooting accuracy, ball handling, and decision-making, increasing the likelihood of turnovers or missed scoring opportunities. Nevertheless, strong intrinsic motivation and self-efficacy can mitigate the effects of mental fatigue, enabling players to maintain confidence and composure under pressure. An adaptive learning orientation allows them to view mistakes as opportunities for development, promoting sustained effort and resilience throughout competitive play [37].

As demonstrated, basketball imposes numerous psychological and physical demands. An athlete’s ability to maintain high-level performance in both domains directly influences in-game effectiveness and, ultimately, the team’s success. Sports science and sports medicine are actively investigating strategies to enhance basketball performance. This has led to the development of training and psychological interventions. However, additional, less conventional techniques may also impact athletic performance. Optimal brain function—as the central organ controlling human cognition and behavior—is essential in sports. Therefore, interventions that directly modulate brain activity may hold promise for improving athletic outcomes. One such method is transcranial direct current stimulation (tDCS), a non-invasive brain stimulation technique that has garnered increasing interest in neuroscience, psychiatry, neurology, and sports science for the past two decades.

tDCS is a non-invasive method that can temporarily alter the activity of specific brain regions [38]. It is painless, safe, well tolerated, and has few to no side effects [39,40]. By applying a weak constant electrical current (1–3 mA) between two electrodes placed over the scalp, tDCS can alter the resting membrane potential and, as a result, increase (via anodal stimulation) or decrease (via cathodal stimulation) the excitability of the targeted brain area [41,42] for up to 90 min post-stimulation. Generally speaking, cathodal stimulation hyperpolarizes neurons, decreasing their likelihood of firing, while anodal stimulation depolarizes neurons, increasing neuronal excitability [43]. The precise effect depends on the orientation of neuronal components within the targeted cortical region. Although tDCS is inherently non-focal due to the current flow between electrodes, localization can be improved by adjusting electrode size [44].

The neuroplastic effects induced by tDCS can be amplified and extended through repeated application. Mechanistically, tDCS activates calcium-dependent synaptic plasticity in glutamatergic neurons via N-methyl-D-aspartate (NMDA) receptors, resulting in either long-term depression (LTD) or long-term potentiation (LTP), depending on intracellular concentrations [45,46]. Delivering 1–2 mA of current for more than 10 min stabilizes these synaptic changes for at least an hour, while even short durations (approximately 3 min) can yield effects that outlast the stimulation period [42]. Furthermore, tDCS modulates neurotransmitters such as glutamate and reduces gamma-aminobutyric acid (GABA) levels, while also influencing brain-derived neurotrophic factor (BDNF), thereby resetting the cortical excitation–inhibition balance [47,48,49,50]. Notably, these alterations are not confined to the stimulated region but may also extend to functionally connected brain networks [51].

Although tDCS is most commonly studied in the treatment of psychiatric and neurological disorders, its application in enhancing athletic performance is an increasingly prominent area of research. Studies have shown that tDCS can improve cycling and running performance [52], overall physical performance in athletes [53], maximal force output, and endurance capacity [54], among other outcomes. Moreover, beneficial effects may be observed even after a single session [55]. The most commonly employed protocol for improving athletic performance involves stimulation of the primary motor cortex. However, it is worth mentioning that the mechanisms of action of tDCS in improving sports performance are poorly understood. Despite numerous findings from literature reviews that tDCS improves sports performance, little is known about how the stimulation works. This is discussed in more detail in Section 5 of this paper.

Regular engagement in athletic training is increasingly recognized as a potent, experience-dependent modulator of neuroplasticity. Longitudinal MRI studies have demonstrated that the acquisition of novel motor skills—such as juggling or complex balance exercises—elicits rapid increases in gray matter density and cortical thickness in the primary motor, premotor, and parietal cortices, accompanied by volumetric expansion of cerebellar lobules involved in sensorimotor prediction [56,57,58,59]. Endurance and skill-based training also remodel white matter microstructure, as evidenced by elevated fractional anisotropy in multiple cortical regions among trained individuals compared to non-athletes, suggesting enhanced myelination and axonal re-organization that facilitate more efficient neural conduction [60,61]. Additionally, aerobic exercise has been shown to upregulate hippocampal BDNF signaling in rats [62], a key molecular mechanism supporting long-term motor memory formation and accelerated decision-making under fatigue.

Conversely, fatigue—a critical factor affecting athletic performance—is associated with altered cortical excitability, as indicated by findings from studies employing repetitive transcranial magnetic stimulation (rTMS), and may disrupt homeostatic neural regulation [63]. Notably, tDCS has also been shown to induce neuroplastic changes via mechanisms partially overlapping with those elicited by physical training. As such, tDCS may serve as an adjunctive technique to augment plasticity processes triggered by sports practice. These neurobiological parallels provide a theoretical rationale for investigating the application of non-invasive brain stimulation to enhance athletic performance, including in basketball.

Due to the numerous physical and mental demands that basketball players are subjected to, this scoping review aims to evaluate whether tDCS can enhance physical and mental performance in basketball players. Given the growing body of research demonstrating its positive effects on numerous parameters critical to athletic success, validating the effectiveness of non-invasive brain stimulation in basketball may popularize its use and support improved performance among competitive athletes. Therefore, this work will seek to answer the question of whether tDCS promises to be a new innovative therapeutic procedure in facilitating the demands placed on basketball players that may potentially affect their performance.

## 2. Methods

This scoping review aims to systematically evaluate the extent to which tDCS improves basketball performance. Strict selection criteria and a comprehensive literature search were employed to ensure the validity and applicability of the presented evidence. The methodology focused on identifying clinical trials that assessed the impact of tDCS on basketball players, in accordance with established protocols for systematic reviews and evidence synthesis, such as the PRISMA guidelines [64]. However, it is important to note that this scoping review does not fully adhere to all PRISMA criteria and instead adopts a scoping approach rather than a completely systematic one. Before the start of the search, a review protocol was entered into the PROSPERO database (CRD420251034988).

### 2.1. Data Sources and Search Strategy

J.Ch. and R.B. conducted an impartial and methodologically sound Internet-based research using a combination of specific keywords, including “basketball” and “tDCS” or “transcranial direct current stimulation”. In February 2025, an extensive search was carried out across several databases—PubMed/Medline, Research Gate, Google Scholar, and Cochrane—focusing on studies published between January 2008 and February 2025.

### 2.2. Study Selection Criteria

To be eligible for inclusion, studies had to be clinical trials published between 2008 and 2025. Each study was required to examine the effects of tDCS on basketball performance. Review articles were excluded from consideration.

### 2.3. Screening Process

A multi-step screening procedure was implemented to ensure the inclusion of relevant studies and the exclusion of those that did not meet the predefined criteria. Two independent reviewers, J.Ch. and R.B., conducted a thorough screening of titles and abstracts.

#### 2.3.1. Title and Abstract Screening

During the initial screening stage, each reviewer assessed the independently available records’ abstracts and titles throughout the first screening process to see if they satisfied the inclusion criteria. The primary focus was on studies that investigated the impact of tDCS on basketball players.

#### 2.3.2. Full-Text Assessment

Papers that passed the initial screening were subjected to a comprehensive full-text assessment. The reviewers verified that each publication was a clinical trial, published between January 2008 and February 2025, written in English and Arabic, and that it met all eligibility requirements.

## 3. Results

**Figure 1:**
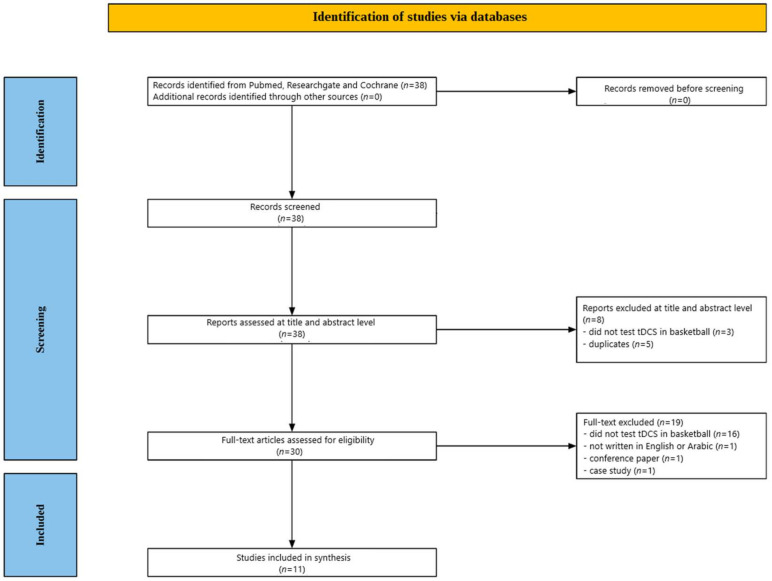
Flow chart depicting the different phases of the scoping review.

Figure 1 provides a summary of the screening process. Of the 38 studies initially identified through database research, eight were excluded based on title and abstract review. Reasons for exclusion included five duplicate entries and three studies that did not examine tDCS in the context of basketball. Following a detailed full-text evaluation of the remaining 30 papers, 16 were excluded for failing to investigate the impact of tDCS on basketball performance, one excluded paper was a case study, one was written in other language than English and Arabic, and one was a conference paper. Ultimately, 11 studies met the inclusion criteria and were incorporated into this review.

### 3.1. Summary of Included Studies

The included studies are summarized in Table 1. In [65], a total of 52 healthy sports students (19 men, 33 women, mean age ≈ 22) were recruited. The experiment followed a double-blind, randomized, sham-controlled, crossover design. Each participant completed two sessions—one involving real tDCS and one sham session—separated by at least 48 h. The order of conditions was determined using sealed-envelope randomization. Each session began with a 10 min standardized warm-up, followed by two basketball-specific performance tests administered both before and after stimulation. For real tDCS, a current of 1 mA was applied for 20 min. The anode was placed over the primary motor cortex of the hemisphere controlling each participant’s dominant hand (C3 or C4 in the 10/20 EEG system), and the cathode was placed over the contralateral supraorbital region. The basketball performance assessments included a 100-shot accuracy test (20 shots from each of five distinct positions on the court) and the Illinois ball-dribbling test (timed completion of a short, cone-marked course at maximum speed while dribbling). For the shooting test, participants received a pass from a partner and attempted each shot using their dominant hand, with the non-dominant hand providing support. The number of successful shots out of 100 was recorded. In the dribbling test, participants performed two valid attempts, with the fastest time retained for analysis. Performance across conditions was analyzed using repeated-measures ANOVAs, with interactions between time (pre-test vs. post-test) and intervention (real vs. sham tDCS) indicating whether stimulation had a statistically significant effect. Additionally, a current flow simulation was performed to visualize the predicted distribution of the electric field within the cortex. The results revealed concentrated effects in dominant motor regions, with some additional influence in non-dominant prefrontal areas.

In [66], researchers investigated whether a single session of tDCS applied over the left dorsolateral prefrontal cortex (lDLPFC) could reduce basketball players’ susceptibility to deceptive maneuvers known as “head fakes”. The study enrolled 50 right-handed adults with limited basketball experience, who were randomly assigned to receive either anodal or cathodal tDCS. The researchers then assessed changes in susceptibility to head-fakes using a purpose-designed laboratory task. The “head-fake” task involved video-based stimuli showing a basketball player facing forward, briefly turning his head (left or right), and then passing the ball either to the same side (congruent) or the opposite side (incongruent). Participants were instructed to respond as quickly and accurately as possible to the actual pass direction, ignoring the player’s gaze direction. The tDCS protocol involved the offline application of a 0.5 mA current for 19 min, meaning stimulation was completed prior to the post-test. In the anodal condition, a small 3 cm × 3 cm electrode (9 cm^2^) was placed at F3 (over the lDLPFC), while a larger 5 cm × 7 cm electrode (35 cm^2^) was positioned on the left deltoid muscle as the reference. In the cathodal condition, the same electrode arrangement was used, but with reversed polarity. The key outcome measure—reaction time in the “head-fake effect”—was defined as the difference in speed (or error rate) between incongruent (fake) and congruent trials. Participants completed two blocks of trials before tDCS (pre-test) and again after tDCS (post-test), and changes in the head-fake effect from pre- to post-test were analyzed.

In [67], 26 skilled male basketball players (mean age ≈ 16.5 years) were randomly assigned to two groups: an experimental group (*n* = 13) that received real tDCS and a sham group (*n* = 13). Prior to the intervention, all participants completed a baseline free-throw test, in which they each performed 10 free throws under standard basketball conditions. A four-point scoring system was used to quantify shooting accuracy: three points were awarded for a clean shot (i.e., no contact with the rim or backboard), two for a successful shot with rim or backboard contact, one point for hitting the rim or backboard without scoring, and zero points for a complete miss. Over three consecutive days, each player followed the same daily routine. First, participants viewed a point-light demonstration of free-throw shooting, created using reflective markers placed on the joints of two national-level basketball players. In a darkened setting, these markers emphasized only the motion path, eliminating visible body outlines to focus attention on joint movements. Immediately after viewing this point-light model, the experimental group underwent 20 min of anodal tDCS (1.5 mA) applied over the left premotor cortex. The reference electrode was positioned on the contralateral (right) arm. Following either the tDCS or sham session, participants proceeded to the basketball court and attempted 10 free throws, with accuracy recorded using a four-point scale.

In [68], 26 skilled male basketball players were randomly assigned to two groups: one receiving real tDCS and the other undergoing a sham condition. Over three consecutive days, each participant followed the same procedure: first, they viewed a point-light model demonstration of a two-point field throw, which highlighted only the essential joint movements of two elite basketball players by using reflective markers in a dark setting. Immediately afterward, the experimental group received 1.5 mA of anodal tDCS over the premotor cortex for 20 min. Both groups then attempted a number of two-point field throws to assess their performance following that day’s intervention. Before the start of this three-day intervention phase, a pre-test was administered, during which participants performed baseline throws. At the conclusion of the three sessions, follow-up assessments were conducted one day later (post-test) and one week later (follow-up), during which participants repeated the same shooting tests. This methodology allowed the researchers to evaluate both within-group and between-group changes in performance, capturing immediate effects during the daily sessions as well as more enduring improvements in the short term (24 h) and slightly longer term (seven days).

The authors of [69] investigated whether tDCS could mitigate the detrimental effects of prolonged cognitive exertion on both cognitive and shooting performance in professional female basketball players. Using a randomized, double-blinded, sham-controlled, crossover design, the study involved eight elite players from a high-level team. These athletes trained 10–12 sessions per week and regularly competed at national and international levels. Participants attended four sessions spaced 72 h apart. The first session introduced the 30 min inhibitory Stroop task, familiarized participants with the tDCS procedure, and included a 5 min active tDCS session to assess individual tolerance. In the second session, a baseline assessment of three-point shooting performance was conducted without prior cognitive load or tDCS. In the third and fourth sessions, players were randomly assigned to receive either anodal tDCS (a-tDCS, 2 mA for 20 min) or sham stimulation during the first 20 min of a 30 min Stroop task. Following the cognitive task, athletes performed the three-point shooting task under both defended and undefended conditions. The tDCS intervention involved electrode placements over the left DLPFC (F3) and right DLPFC (F4), with stimulation set at 2 mA for 20 min. The prolonged cognitive task (PCT) comprised a 30 min modified Stroop task involving only incongruent trials to test inhibitory control. Response time (RT) and accuracy were recorded every five minutes. To assess cognitive workload and motivation, players completed the NASA Task Load Index (NASA-TLX) and reported motivation levels before and after the task using the Success Motivation and Intrinsic Motivation scales. Following the Stroop task, participants attempted 10 successful three-point shots under both defended and undefended scenarios, with the total number of attempts recorded. Finally, perceived exertion was measured using the CR-10 Borg scale.

In [70], 13 trained male basketball players (mean age ≈ 20 years) participated in a single-blind, randomized crossover design. Each player undertook two separate trials—one involving real tDCS and the other with a sham condition—spaced approximately 7–10 days apart. The tDCS intervention lasted 20 min at an intensity of 2 mA, with electrodes positioned at Cz, C5, and C6 according to the 10–20 EEG system, targeting the motor cortices on both hemispheres with anodal stimulation. Upbeat music was played through the headset to divert participants’ attention and maintain effective blinding. Following the 20-minute stimulation or sham condition, participants rested for 10 min while heart rate monitors were fitted, and resting heart rate was recorded. A dynamic warm-up then commenced, including mobility drills such as squats, lunges, high-knee runs, side steps, short sprints, and concluding with squat jumps. Next, participants performed three trials of a standard countermovement jump (CMJ), each separated by about 30 s, followed by three trials of a weighted CMJ holding 20 kg (10 kg in each hand). Jump height was measured using a Gymaware device attached at the waist. The average heights for both standard and weighted CMJs were recorded, and the ratio between them was used as an indicator of lower-body power and maximal strength. Immediately following the jumping tests, participants completed 40 repeated 15 m shuttle sprints using a 1:4 work-to-rest ratio. Each sprint, which lasted approximately 2–3 s, was followed by 8–12 s of rest, managed with the help of a Witty timing system. This system tracked sprint completion times and initiated a five-second countdown prior to each subsequent sprint. Spring performance was monitored across intervals (e.g., after sprints 1–10, 11–20, 21–30, and 31–40). Heart rate was recorded before and after selected sprints, and ratings of perceived exertion (RPE) were noted at baseline (0) and following the 10th, 20th, 30th, and 40th sprints. A fatigue index was calculated by comparing the ideal total sprint time (fastest sprint × 40) with the actual cumulative sprint time. Data distribution was verified using Shapiro–Wilk tests. Two-way repeated-measures ANOVA was employed to assess interactions between condition (tDCS vs. sham) and time (across the repeated sprints or jump tasks). Where significant effects emerged, paired *t*-tests were used to compare jump height, sprint time, heart rate, RPE, and fatigue index between the tDCS and sham conditions.

In [71], 45 female university students, each identified as a novice at basketball free-throw shooting, were selected via purposive sampling, which excluded individuals with intermediate or advanced accuracy based on a preliminary test. Participants were randomly assigned to one of three groups (*n* = 15 per group): (1) tDCS targeting the motor cortex (C3 anode, Fp2 cathode), (2) tDCS targeting the visual cortex (Oz anode, Cz cathode), and (3) sham tDCS. All participants completed a pre-test consisting of 15 free throws. Over six consecutive days, each group underwent its designated stimulation protocol for approximately 15 min, immediately followed by a practice session of 15 free throws. The motor cortex group received 1.5 mA anodal stimulation at C3 with the reference electrode at Fp2, while the visual cortex group received 1.5 mA at Oz with the reference at Cz. On the sixth day, all participants performed a post-test of 15 free throws. Subsequently, they returned 1 week later for a short-term retention test and again 21 days later for a long-term retention test, each consisting of 15 free throws. Free-throw attempts were scored using a four-point scale (three points for a clean make, two if the shot hit the rim of the backboard and went in, one if it hit the rim of the backboard but missed, and zero points for misses with no contact).

In [72], 18 male basketball players were recruited using convenience sampling and randomly allocated into two groups (experimental vs. control), each consisting of nine participants. Initially, each player’s baseline mental fatigue was assessed using the Self-Evaluation of Mental Fatigue Scale (a visual analog scale ranging from 0 to 10). Participants then completed a mental fatigue induction protocol, which included a Stroop test (delivered via software) combined with a basic arithmetic task. Immediately after completing these tasks, participants re-evaluated their mental fatigue using the same scale to verify that they had reached a “tired” state, indicated by an above-average mental fatigue score. Once mental fatigue was successfully induced, pre-tests were administered, including both a fatigue assessment (the same 0–10 scale) and basketball performance evaluation using the AAHPERD three-point shot test. In the performance test, participants attempted standardized three-point shot tests, with accuracy scores recorded. After the pre-test phase, the experimental group received tDCS at 1.5 mA for 25 min. The anodal electrode was placed over the DLPFC on the left side (F3), while the cathodal electrode was positioned on the right supraorbital area.

In another experiment [73], researchers employed a randomized, double-blind, counterbalanced crossover design that included five experimental sessions: one familiarization session, two baseline visits, and two experimental conditions, separated by a one-week washout period. Twenty professional male basketball players (aged 18–31, M = 24.77 ± 4.2 years) were recruited. All participants had at least three years of national-level experience, trained between five and six times per week (mean 14.83 h), and had an average of 6.9 years of professional basketball experience. To induce mental fatigue, participants engaged in a 60 min sports-based video game session using NBA Live 19 (EA Sports) on a PlayStation 4 connected to a 48-inch television. Previous research has indicated that prolonged gameplay in such simulations increases frontal midline theta-wave activity, indicative of cognitive load. The game was selected due to its demands for sustained attention, cognitive flexibility, and rapid decision-making, all of which contribute to mental fatigue. During the experimental condition, the anodal electrode was placed over the motion-sensitive middle temporal area (CP5), while the cathodal electrode was positioned over the visual cortex (Oz), according to the 10–10 EEG international system. The stimulation protocol involved a current of 2.0 mA applied for 30 min via rubber conductive electrodes (5 × 5 cm; 0.08 mA/cm^2^). Multiple outcome measures were collected throughout the study. Subjective mental fatigue was assessed using a 100 mm Visual Analog Scale (VAS) with endpoints from 0 (none at all) to 100 (maximum fatigue). Motivation was evaluated using a similar 100 mm VAS, administered before and after the mental fatigue task. Visual search behavior was recorded using an Eye Tracking-XG device (Applied Science Laboratories) operating at a sampling frequency of 60 Hz, with an additional 25 Hz camera capturing gameplay scenarios. Data were analyzed frame by frame using Gaze Tracker and Kinovea software, from which fixation-related metrics (number and duration of fixations) were extracted. Visuomotor skill was evaluated using the Fitlight Training System. Participants were required to respond to LED stimuli while continuously bouncing a basketball, with performance measured through response time and accuracy. The basketball decision-making task involved viewing custom-recorded five-on-five game scenarios filmed from a first-person perspective using a GoPro Hero 3. Players were required to select offensive (dribble, pass, shoot) or defensive (block, steal, move) actions in response to each scenario. Decision-making accuracy and response time were recorded. For statistical analysis, a two-way repeated-measures ANOVA was conducted to compare subjective mental fatigue and motivation, with group (a-tDCS vs. sham) and time (precognitive vs. postcognitive task) as fixed factors. Percentage change (Δ%) was calculated using the formula Δ% = [(post − pre)/pre] × 100. Additional two-way ANOVAs were conducted to examine group × time interactions for eye-tracking measures (eyeblink duration and pupil diameter) across four 15 min intervals, as well as for visuomotor and decision-making outcomes (accuracy and response time) at baseline and post-intervention.

The authors of [74] employed a semi-experimental design with pre-test, post-test, and follow-up assessments at 7 and 21 days to investigate the combined effect of tDCS and observational learning on basketball free-throw skill acquisition and retention. Thirty novice female basketball players were selected through convenience sampling and randomly assigned to one of two experimental groups: (1) the tDCS with model observation group or (2) the sham (placebo) stimulation with model observation group. All participants were right-handed and demonstrated low to moderate free-throw proficiency, as determined by a pre-study screening test. The experiment lasted five consecutive days and consisted of three phases. During the pre-test phase, all participants completed 15 basketball free-throw trials to establish a performance baseline. In the intervention phase, participants in the tDCS group received 15 min of anodal stimulation over the motor cortex (C3 anode, Fp2 cathode) while watching a skilled model demonstrate free-throw performance. Immediately following stimulation, they attempted 15 free throws. After completing five days of intervention, participants entered the post-test phase, where they again performed 15 free-throw attempts. To evaluate skill retention, short-term and long-term retention tests were conducted at 7 and 21 days post-intervention, respectively, using the same free-throw protocol. The tDCS current was set at 1.5 mA for 15 min. Free-throw performance was the primary outcome, assessed using a standardized scoring system at pre-test, post-test, and retention intervals. A two-way repeated-measures ANOVA was used to analyze the effects of time (pre-test, post-test, short-term retention, and long-term retention) and group (tDCS vs. sham), with post-hoc tests conducted to determine significant differences between conditions.

The authors of [75] conducted a double-blind randomized clinical trial designed to compare the effects of mental imagery and tDCS on basketball shooting skills in non-elite players. The study included 36 male and female participants aged from 18 to 25, all with no more than three years of basketball experience. Participants were randomly assigned to one of three groups: a mental imagery group, a tDCS stimulation group, or a control group that continued regular basketball training without additional intervention. Mental imagery ability was assessed using the MIQ-3 questionnaire, and only individuals with moderate to high scores were included. The intervention phase lasted six weeks, with two sessions per week, totaling 12 sessions. Each session followed a standardized structure: participants completed their assigned intervention and then performed a basketball shooting task. In the mental imagery group, participants engaged in a guided visualization session in which they visualized the shooting movement in a quiet and controlled environment with their eyes closed. This visualization process lasted seven minutes. In the tDCS group, participants received 20 min of anodal stimulation at 2 mA using 5 cm × 7 cm electrodes, with the anode placed over M1 (left hemisphere) and the cathode over the right supraorbital area. Shooting performance was evaluated using a modified version of the AAHPERD basketball shooting test. Each participant attempted 20 free throws from a distance of 6.25 m, and shot accuracy was recorded. Performance was assessed at three time points: before the intervention (pre-test), immediately after the six-week intervention (post-test), and one month later to assess skill retention (retention test). Statistical analysis was conducted using repeated-measures ANOVA to examine within-group and between-group differences across the three time points. Bonferroni post-hoc tests were used to determine pairwise differences. Effect sizes (η^2^) were calculated to evaluate the magnitude of intervention effects. This design enabled a rigorous and controlled comparison of the impact of mental imagery and tDCS on basketball shooting performance and long-term skill retention.

### 3.2. Impact on Shooting Performance

Across the eight studies investigating the effects of anodal tDCS on basketball shooting, a generally consistent pattern of performance enhancement emerged, though with several important caveats. In [65], administering 1 mA of motor cortex stimulation during practice resulted in a statistically significant increase in successful shots from pre- to post-test, as reflected by a time-by-condition interaction (F_1,51_ = 5.60, *p* = 0.022) and a small-to-moderate effect size (d = 0.45); no such improvement was observed in the sham group.

Building on these findings, ref. [66] monitored performance over three training sessions followed by two delayed assessments. Although neither the real-tDCS nor the sham group improved during the intervention phase (repeated-measures ANOVA, *p* = 0.337) and between-group differences were initially non-significant (*p* = 0.081), a marked divergence emerged 24 h after the final session and widened further at one-week follow-up. A significant group-by-time interaction (F_2,48_ = 6.15, *p* = 0.004) indicated that pairing anodal stimulation with point-light model observation produced delayed, yet substantial, gains in free-throw accuracy (*p* < 0.001 for both post-test and follow-up within-group contrasts), whereas the sham group showed no meaningful change.

A similar pattern was observed in [68]. During the three active sessions, both groups improved comparably (tDCS: ≈16.3 → 19.0; sham ≈ 17.8 → 18.2), with no significant between-group difference. However, at the one-day post-test, the tDCS group outperformed controls (21.23 ± 3.41 vs. 17.84 ± 5.98, *p* < 0.05); and this performance gap widened further at the seven-day follow-up (22.76 ± 4.55 vs. 17.73 ± 5.59, *p* < 0.05), again suggesting a post-intervention consolidation effect.

Not all protocols yielded positive results. Reference [69], which employed an on-court, defended/undefended task, found no between-condition differences immediately post-intervention or after a brief rest (all *p* ≈ 0.65). This null finding may reflect limiting factors such as low stimulation intensity (1 mA for only 10 min), suboptimal electrode montage, or task complexity that may have attenuated neuromodulatory effects.

In contrast, ref. [71] compared motor cortex and visual cortex stimulation with sham across six days. Both active conditions significantly outperformed sham at post-test, with motor cortex tDCS producing the largest improvement (F = 16.91, *p* = 0.0001, η^2^ = 0.547) and sustaining superiority at one- and three-week follow-ups, suggesting both site-specific efficacy and durable neuroplastic effects.

Similarly, ref. [72] demonstrated that combining anodal tDCS with standard training enhanced three-point accuracy more than practice alone (tDCS: 29.7 ± 4.5 to 36.8 ± 4.5, control: 30.4 ± 2.0 to 32.7 ± 2.0 (*t*(16) = 2.52, *p* = 0.02).

Finally, refs. [74,75] reported especially pronounced effects when tDCS was combined with action-model observation or mental imagery. In [66], stimulation produced a large effect size (η^2^ = 0.615 (F = 22.33, *p* = 0.0001) and outperformed sham at immediate, 7-day, and 21-day follow-ups (*p* ≤ 0.006). Similarly, in [75], although all groups improved, the tDCS group showed demonstrated the steepest performance trajectory (F = 114.03, *p* = 0.0001), outperforming both imagery and control groups at all post-intervention time points (*p* ≤ 0.006).

### 3.3. Impact on Dribbling Performance

In [65], repeated-measures ANOVA revealed a significant time × intervention interaction (F_1,51_ = 4.53; *p* = 0.038), with participants reducing their dribbling times more effectively under real tDCS than under sham stimulation (d = 0.40).

### 3.4. Impact on Reaction Time

The results from [67] showed that, in the anodal tDCS group, incongruent reaction times (RTs) dropped from about 397 ms at the pre-test to 366 ms at the post-test (compared to 324 ms and 307 ms, respectively, for congruent trials), reducing the head-fake effect by roughly 14 ms (t(24) = 2.28, *p* = 0.03). By contrast, the cathodal group improved by only about 2 ms (t(24) = 0.72, *p* = 0.48). A 2 × 2 repeated-measures ANOVA confirmed a significant overall improvement (*p* < 0.05), although the session × tDCS interaction only approached significance (*p* = 0.1). Nevertheless, within-group comparisons showed that anodal stimulation led to a clear and reliable reduction in the head-fake effect, while cathodal stimulation did not. Accuracy remained consistently high (approximately 97–99%) across all conditions and sessions, with no meaningful variation.

### 3.5. Impact on Jump Performance

In [70], during the countermovement jump (CMJ) test, the players in the real tDCS condition recorded a significantly higher jump height than those in the sham condition (*p* = 0.04), although the weighted CMJ results did not differ statistically (*p* = 0.427).

### 3.6. Impact on Sprint Times

In [70], during 40 repeated 15 m shuttle sprints, participants in the real tDCS group showed a distinct advantage beginning around the 20th sprint. Specifically, sprint times in the 21st–30th and 31st–40th intervals were significantly shorter for the tDCS group compared to the sham group (*p* = 0.018 and *p* = 0.014, respectively), resulting in a significantly lower overall average sprint time across all 40 runs (*p* = 0.016).

### 3.7. Impact on Fatigue

Across the four studies that examined exertion- and fatigue-related outcomes, anodal tDCS produced limited and inconsistent effects. In terms of perceived exertion, no statistically significant differences were observed. In [69], mean session RPE values remained in the “very light” range and did not differ significantly across real, sham, and baseline conditions (2.6 ± 0.7, 3.1 ± 0.6, and 3.0 ± 0.7, respectively; *p* = 0.391), Similarly in [70], both heart rate and RPE scores were comparable between stimulation conditions. However, ref. [70] reported a significantly lower physiological fatigue index under tDCS (*p* = 0.009), indicating reduced speed decrement across repeated sprints, despite no corresponding change in subjective effort.

Self-reported mental fatigue increased substantially following task exposure in the remaining two studies. In [72], scores rose from approximately 3.7 to 7.1 in the tDCS group and from 3.7 to 6.2 in the control group. Both within-group increases were significant (*p* = 0.001), though the between-group post-test difference was not (*p* = 0.11). In study [73], mental fatigue also increased in both conditions, but the rise was significantly greater in the sham group compared to the tDCS group (*p* < 0.05); motivation ratings remained stable across time points and condition.

In summary, perceived exertion (RPE) and heart rate were unaffected in two studies; one trial reported reduced physiological fatigue with tDCS; and mental fatigue outcomes were mixed—non-significant in one case and significantly attenuated in another—while motivation was unchanged.

### 3.8. Impact on Visuomotor Skills

In [73], post-intervention response time for the visuomotor skill task was significantly slower in the sham condition compared to the a-tDCS condition (*p* < 0.05), indicating that a-tDCS helped maintain response efficiency under mental fatigue. However, no significant differences were found in visuomotor skill accuracy between conditions (*p* = 0.76), suggesting that a-tDCS preserved response speed but did not enhance accuracy.

### 3.9. Impact on Decision-Making

In [73], both response time and accuracy in the basketball decision-making tasks, were significantly impaired in the sham condition compared to the a-tDCS condition (*p* < 0.05). The effect size for response time was very large (η^2^*p* = 0.25), indicating a strong protective effect of a-tDCS against cognitive fatigue-induced declines in decision-making efficiency. Accuracy also showed a large effect size (η^2^*p* = 0.11), confirming that players in the a-tDCS group made better decisions under mental fatigue.

### 3.10. Impact on Visual Search Behavior

In [73], regarding visual search behavior, the number of fixations was significantly lower in the sham condition compared to the a-tDCS condition (*p* < 0.05), indicating that mental fatigue affected visual scanning strategies. Additionally, fixation duration was significantly longer in the sham condition than in the a-tDCS group (*p* < 0.05), suggesting that mentally fatigued players in the sham condition engaged in prolonged fixations, possibly due to reduced efficiency in visual information processing.

### 3.11. Impact on Inhibitory Control

Results from [69] showed that response time improved over the 30 min Stroop task; however, no significant differences were observed between the a-tDCS and sham conditions (*p* > 0.05). The mean RT did not differ significantly between groups (a-tDCS: 830 ± 186 ms, sham: 897 ± 164 ms, *p* = 0.373), and accuracy remained high in both conditions with no significant difference (a-tDCS: 93 ± 8%, sham: 94 ± 4%, *p* = 0.452). No condition × time interaction effects were found for either RT (*p* = 0.291 for RT, *p* = 0.759 for accuracy).

### 3.12. Impact on Cognitive Workload and Motivation

In [69], motivation levels remained stable before and after the Stroop task, with no statistically significant differences between the a-tDCS and sham conditions (*p* > 0.05). The NASA-TLX scores confirmed a high cognitive workload overall, but again, no significant differences emerged between the conditions. Mental demand scores were nearly identical (83.8 ± 19.8 for a-tDCS and 83.7 ± 21.8 for sham; *p* = 0.989). Similarly, no significant differences were found across other NASA-TLX subscales; physical demand (a-tDCS: 15.5 ± 13.0, sham: 18.7 ± 15.0, *p* = 0.646), temporal demand (a-tDCS: 45.0 ± 26.7, sham: 36.2 ± 33.8, *p* = 0.560), performance self-assessment (a-tDCS: 61.6 ± 21.3, sham: 63.1 ± 16.2, *p* = 0.877), effort (a-tDCS: 65.5 ± 17.5, sham: 71.8 ± 18.5, *p* = 0.481), and frustration (a-tDCS: 43.8 ± 28.9, sham: 50.0 ± 32.9, *p* = 0.689).

### 3.13. Comparative Insight and Methodological Appraisal of the tDCS Evidence in Basketball

Across the 11 studies investigating tDCS in basketball players, the evidence suggests a promising but methodologically limited field. Sample sizes ranged from eight world-class professionals [69] to forty-five novices [71], with adolescent national hopefuls [67,68] and experienced adult competitors [65,66,70,72,73,74,75] representing intermediate groups. While this diversity enhances ecological validity, it also introduces substantial heterogeneity in training age, skill repertoire, and neuroplastic potential, thereby complicating the interpretation and generalization of effect sizes.

Ecological validity varied considerably. Only two studies assessed performance under realistic decision-making or defensive pressure [69,73]; the majority relied on isolated free throws, point-light video practice, or laboratory-based shuttle sprints, which differ markedly from in-game conditions.

Internal validity showed moderate strength. Eight studies employed sealed-envelope or computer-generated randomization with sham controls [65,66,67,68,69,71,73,74], but blinding procedures were often suboptimal. Four studies were single-blind or omitted any blinding validation [68,70,71,72], and two used inadequate comparators such as “cathodal” or vaguely defined “artificial” currents [66,71]. Only one study reported an a priori sample size calculation [65], and several positive findings were based on groups of 10–15 participants, raising concerns about type I error and effect size inflation.

Stimulation parameters varied widely. Current intensities ranged from 0.5 mA over the left DLPFC [66] to 2 mA bilaterally over M1 [70]. Target areas included the primary motor cortex [65,71,74,75], premotor cortex [67,68], dorsolateral prefrontal cortex [69,72,73], and motion-sensitive middle-temporal areas [73]. Reference electrode placements included the supra-orbital region, deltoid, vertex, and occipital cortex. Only three studies employed electric-field modeling [65,73,75], and none confirmed cortical engagement using neurophysiological tools such as TMS-MEP or EEG, leaving the effective dose uncertain.

Outcome measures were poorly standardized. Shooting accuracy, the most frequently assessed metric (eight studies), was measured using raw percentage in one study [65] and as zero-to-three or zero-to-four ordinal scales in the rest [67,68,71,74,75]. Dribbling time was assessed in only one study [65]; while decision-making, visual search, and visuomotor speed were measured in a single mental fatigue protocol [73]. No study reported in-game metrics such as field goal percentage, turnovers, or player efficiency. Although repeated-measures ANOVA was the most common analytic approach, multiplicity corrections were rarely applied, and selective reporting cannot be ruled out.

Despite this heterogeneity, several patterns emerge. When 1.5–2 mA of anodal stimulation was applied to the primary motor cortex—particularly when combined with physical practice or observational learning—robust and durable improvements in free-throw or two-point shooting accuracy (partial-η^2^ ≈ 0.55–0.62), lasting up to three weeks post-intervention [68,71,74,75]. Premotor cortex stimulation appeared to support delayed performance consolidation in skilled youth rather than immediate boost, with benefits surfacing 24 h post-intervention [67,68]. By contrast, DLPFC-targeted tDCS did not enhance baseline performance but mitigated mental fatigue-induced declines in visuomotor reaction and decision accuracy [69,72,73]. Null results tended to occur in studies with small, elite samples exposed to demanding dual-task paradigms, suggesting a potential ceiling effect for prefrontal tDCS under high-performance conditions [69].

In general, these findings suggest that well-targeted anodal tDCS may enhance motor-skill acquisition and attenuate fatigue-related cognitive decline in basketball. However, the overall certainty remains low due to small sample sizes, heterogeneous montages, inconsistent sham protocols, and non-standardized outcomes [65,66,67,68,69,70,71,72,73,74,75]. Advancing this field will require adequately powered, multi-center RCTs with CONSORT-compliant reporting, FIBA-relevant standardized endpoints, neurophysiological dose verification, and factorial designs capable of disentangling the effects of adjunct learning strategies from stimulation alone. Until such evidence accumulates, tDCS should be considered a promising but still experimental approach to performance enhancement in basketball.

### 3.14. Risk of Bias Assessment

Of the eleven available studies, only one met the criteria for low risk of bias, while six were classified as high risk and four as having some concerns.

The most rigorous study was a double-blind, sham-controlled crossover trial conducted with elite female athletes [69]. It employed computer-based randomization with sealed-envelope concealment, verified blinding credibility, preregistered its analysis plan, and used objective endpoints (video-timed Stroop reaction times, electronically scored shots). Attrition was minimal, and adverse events were systematically reported.

A second crossover study involving adult athletes [65] met most low-risk criteria, including sealed-envelope randomization, identical ramp-up/down sham procedures, and objective scoring, with attrition below 5%. However, the absence of preregistration introduced uncertainty regarding selective reporting, resulting in a rating of “some concerns”. A third crossover involving professional men [73] also featured robust concealment and objective measurements (eye tracking, Fitlight), but lacked preregistration and did not apply statistical correction for multiple comparisons, again warranting “some concerns”.

Several studies were classified as high risk due to insufficient blinding. The “head-fake” study [66] used cathodal stimulation as the control, which is a condition that produces unmistakably different scalp sensations; no blinding check was reported, making performance and detection bias likely. The two premotor cortex studies involving adolescents [67,68] employed single-blind procedures in which assessors knew group allocation, and scoring was done with a subjective zero-to-three accuracy rubric, compounding the risk of deviation and measurement bias; neither study registered an analysis plan, and both reported only statistically significant outcomes, increasing the risk of bias due to selective reporting.

Similar issues were present in the novice free-throw study [71] and the mental fatigue experiment [72], both of which used vaguely described “artificial” sham conditions without sensory blinding, omitted credibility checks, relied on manual scoring, and selectively reported positive outcomes. Methodological shortcomings also affected [70], which failed to describe randomization procedures, employed unblinded assessors, and introduced music during active stimulation—a potential co-intervention absent in the sham group. Reference [74], although stronger in allocation and blinding procedures, reported tingling only in the active group and excluded sham dropouts without data imputation, increasing attrition and reporting bias.

Overall, the current evidence base is undermined by inconsistent sham credibility, underpowered samples (seven studies included ≤15 participants per arm), reliance on subjective outcome measures, and a general absence of preregistered protocols [65,66,67,68,69,70,71,72,73,74,75]. Until future trials adopt rigorous allocation procedures, validated blinding assessments, objective metrics, and full prospective registration, claims regarding the efficacy of tDCS in basketball performance should be viewed as preliminary.

## 4. Discussion

The collective findings from these studies illuminate several nuanced ways in which tDCS can influence various aspects of basketball performance. Overall, the evidence suggests that tDCS may enhance certain motor skills—especially shooting accuracy, dribbling ability, sprint performance, and decision-making—although the magnitude and consistency of these effects vary depending on factors such as stimulation site, intensity, duration, participants’ skill level, and whether the intervention is combined with other methods (e.g., observational learning, cognitive tasks). However, it should be noted that the studies included in this review are exploratory in nature, vary in the paradigms used, and have small study samples of athletes. Furthermore,, primary outcomes are seldom prespecified or justified via power analyses, diverse montages/doses suggest parameter-finding rather than hypothesis-confirmation, and the absence of physiological verification mark them as pilot studies probing feasibility/effect direction. For this reason, the interpretation of results should be treated with caution.

### 4.1. Shooting Performance

Several studies have reported positive, though sometimes delayed, improvements in shooting accuracy. For instance, refs. [65,66,68,71,72,74,75] all found that real tDCS led to greater improvements in shooting performance compared to sham conditions, whereas [69] reported no significant difference. Interestingly, some studies that did not observe immediate effects (e.g., [68]) later identified pronounced benefits at follow-up assessments (one day or more after stimulation), suggesting a potential time lag in the consolidation or retention processes facilitated by tDCS.

Moreover, the placement of electrodes seems to be a critical factor: stimulation over the motor cortex was consistently associated with the most pronounced shooting gains ([65,71,74,75]), whereas targeting other cortical areas such as the dorsolateral prefrontal cortex (DLPFC) [69] or visual cortex [71] yielded mixed or less substantial effects.

A consistent theme across the literature is that anodal stimulation may facilitate neural mechanisms that underlie skill acquisition and motor memory consolidation, especially when coupled with dedicated practice methods (e.g., point-light modeling, observational learning, or physical drills). In several studies, participants displayed little to no immediate improvement during intervention sessions but demonstrated significant performance gains afterward, suggesting that tDCS may primarily enhance later stages of learning—such as memory consolidation—rather than producing immediate improvements in motor execution.

Shooting performance is influenced by various physiological and neuromotor parameters, one of which is upper limb function. A recent systematic review and meta-analysis showed that when compared to sham stimulation, tDCS produced statistically significant improvements in reaction time (RT) (effect size −0.01; 95% CI from −0.02 to 0.001, *p* = 0.03) and execution time (ET) (effect size −0.03; 95% CI from −0.05 to −0.01, *p* = 0.017). Additionally, increased force output (effect size 0.10; 95% CI from 0.08 to 0.13, *p* < 0.001) and a trend toward improved time to task failure (TTF) were noted in physical activity tasks [76].

Shooting performance is the most important parameter influencing basketball results; it determines whether a player is effective and accurate in shooting. Although tDCS appears to be a promising method for improving shooting performance in basketball players, studies measuring the impact on this parameter have numerous limitations. Firstly, nearly all trials have been under-subscribed: most have enrolled a dozen or so participants per arm and offered no a priori power analysis. Small samples magnify random noise and exaggerate any true effect, while cross-over designs with washouts of just 48 h risk carry-over learning that could masquerade as a stimulation benefit. Blinding and control conditions have also been inconsistent. Some “positive” studies have contrasted anodal with cathodal or “no-tDCS” arms rather than using a credible sham, and none have verified whether athletes could guess which condition they had received. Because tDCS often tingles and commercial headsets look and feel different from sponge pads, expectancy or Hawthorne effects could easily inflate post-stimulation accuracy. Intervention parameters have been heterogeneous, ranging from 0.5 mA to 2 mA, with electrode areas of 9–35 cm^2^, and targets that have included the primary motor cortex, premotor cortex, dorsolateral prefrontal cortex, visual cortex, and middle-temporal motion area. With such variation—and no replication of any single protocol—positive findings cannot simply be pooled or generalized. Outcome measures have been equally diverse. Some authors have counted raw makes; others have used four-point ordinal scales or the number of attempts needed to sink 10 baskets. Shot distances varied (free throw, two-point, three-point), and reliability data for these tests were rarely reported. Without a common, validated metric, it is unclear whether the reported gains—often just two to four extra makes—exceed ordinary day-to-day fluctuation. Practice sessions further confound interpretation. Many studies have combined stimulation with model observation or multiple days of shooting practice, particularly in novices whose performance naturally improves steeply with repetition. Because sham groups did not always receive an equivalent practice or modelling dose, any additional progress in the tDCS arm may reflect extra training rather than neurostimulation. Indeed, the largest effects consistently appeared in beginners, while the sole study in elite professionals found no shooting advantage at all, suggesting a ceiling beyond which tDCS offers little incremental help. Where benefits have been observed, they often emerged 24 h or a week after stimulation, not during the intervention itself. Such delays could signal genuine plastic changes, or they could simply reflect routine consolidation of newly practiced skills—again, something the control condition may share to a lesser degree. Compounding these problems, most papers tested multiple endpoints (accuracy, reaction time, retention, kinematics) but applied no family-wise error correction, so some of the statistically “significant” results may have been chance findings. Ecological validity was also limited. Laboratory head-fake reaction time tasks or 100-shot drills omit the stressors that define real play—defensive pressure, crowd noise, fatigue, game stakes—so the question of whether tDCS-induced gains would survive in competition is unknown. Finally, none of the shooting studies verified that the target cortex was actually modulated; measures such as motor-evoked potentials, EEG, or motion-capture kinematics were absent. Without physiological confirmation, any causal links between cortical excitation, biomechanical change, and improved accuracy remain speculative.

### 4.2. Other Basketball-Specific Skills (Dribbling, Head-Fake Susceptibility, Decision-Making)

Research on tDCS and basketball skills other than shooting is so sparse and methodologically uneven that any firm conclusion remains premature, despite promising initial results. Study [65] found that dribbling performance improved more under real tDCS than under sham, supporting the notion that motor cortex stimulation facilitates automated motor skills. It should be noted that these results can be easily confounded by practice: because every athlete repeated the drill four times in 48 h, ordinary learning or lingering carry-over could explain the marginal gain. No retention test, no kinematic analysis, and no data from trained ball-handlers were provided, so the practical value of that fractional timesaving on a cone course is unclear. The authors of [66] investigated players’ susceptibility to the classic “head-fake” maneuver and found that anodal stimulation of the left DLPFC reduced vulnerability to deceptive gaze cues. Although the session × tDCS interaction did not reach statistical significance, within-group comparisons indicated that anodal stimulation produced a small but meaningful reduction in reaction time differences between congruent and incongruent (fake) trials. Because cathodal stimulation can itself impair performance, the observed difference may have reflected hindrance in the control arm more than facilitation in the experimental arm. Moreover, the task was a key-press laboratory surrogate that omitted movement execution, spatial pressure, and fatigue; accuracy was already near the ceiling; and the critical session-by-stimulation interaction barely missed statistical significance, rendering the finding fragile at best.

The most elaborate exploration concerns decision-making and cognitive resilience under mental fatigue. In a counter-balanced crossover study of 20 professional men, researchers delivered 2 mA anodal current between the motion-sensitive middle-temporal area (CP5) and the occipital pole while players engaged in a 60-min basketball video game known to induce cognitive fatigue [73]. Compared with sham, tDCS blunted the rise in subjective fatigue and preserved visuomotor response times, visual-search efficiency, and video-based offensive/defensive decision accuracy, with moderate-to-large partial-eta-squared values. Yet the montage was novel and mechanistically speculative; more than 30 outcomes were tested without family-wise error correction, so some significant *p*-values may have been false positives; and all assessments were video- or light-board-based, not in live play. The intervention protected performance from deteriorating rather than enhancing baseline ability, and the study did not track whether preserved cognitive metrics translated into better scrimmage or match statistics.

Across all three domains, several cross-cutting weaknesses recur: every skill rests on a single unreplicated study; outcome tasks differ so much that results cannot be pooled; none of the investigations verified cortical engagement with TMS, EEG or neuro-imaging; blinding integrity was never checked, raising expectancy–effect concerns; and, most critically, no study linked laboratory or drill improvements to objective in-game performance. Consequently, the tentative gains in dribbling speed, the tiny reaction time advantage against head fakes, and the fatigue-buffering effects on decision-making must all be regarded as hypothesis-generating rather than evidence-based. Coaches and athletes should therefore treat tDCS for these basketball-specific skills as an intriguing possibility that awaits rigorous, preregistered, adequately powered replication under real-world conditions before it can be considered a reliable performance tool.

### 4.3. Physical Performance (Jumping and Sprinting)

The authors of [70] offered insight into whether tDCS could enhance explosive movements by demonstrating that participants in the real tDCS condition achieved significantly higher countermovement jump (CMJ) heights and better shuttle-sprint performance during repeated sprints. These findings support the idea that motor cortex stimulation might facilitate neuromuscular recruitment patterns and delay the onset of performance decline, particularly in repeated-effort tasks. Although weighted CMJ heights in [70] showed no significant differences, the improvements in unweighted CMJ and sprint performance underscore a potential role for tDCS in enhancing short-duration, high-intensity movements—possibly via increased motor unit activation or improved fatigue resistance.

A systematic review and meta-analysis of the sub-group analysis showed that anodal-tDCS improved time to exhaustion (TTE) (standardized mean difference (SMD) = 0.37; 90% CI = 0.13, 0.61; *p* = 0.01) but not sprint performance (SMD = 0.19; 90% CI = −0.23, 0.60; *p* = 0.46) or exercise time to task failure (ETT) (SMD = 0.00; 90% CI = −0.29, 0.30; *p* = 1.00). According to this meta-analysis, the effect of anodal-tDCS may be task-dependent in whole-body dynamic activities such as cycling and running. While it improves TTE performance, it does not appear to enhance spring or ETT outcomes. Most protocols included in the meta-analysis involved stimulation of M1 [52].

### 4.4. Influence of Mental Fatigue and Cognitive Tasks

While several studies have suggested that tDCS can mitigate declines in performance due to cognitive or mental fatigue, refs. [69] and parts of [72] found little difference between real and sham stimulation in reducing feelings of mental fatigue. However, ref. [69] also reported that motivation levels remained unchanged, and NASA-TLX scores reflecting perceived cognitive workload were similarly high across stimulation conditions. This discrepancy likely resulted from differences in study design, electrode placement, or the nature of the cognitive task used to induce fatigue (e.g., Stroop task vs. more immersive or gaming-based cognitive tasks). Moreover, the included studies used only a single stimulation, which may be insufficient to influence the change of this parameter. For this reason, tDCS cannot be recommended as an effective tool for reducing fatigue. Further studies are needed to test the effect of tDCS on fatigue in basketball players, but after a larger number of stimulation sessions, e.g., after 10 sessions.

Nevertheless, results from a systematic review and meta-analysis showed that active a-tDCS considerably reduced ratings of perceived exertion (RPE). When applied over M1 or DLPFC, the a-tDCS may reduce the RPE. The accuracy of RPE measurements may be improved by using standardized scales such as the OMNI scale and Borg’s 6–20 scale [77]. Moreover, ref. [73] provides clear evidence that tDCS can help maintain visuomotor speed and decision-making accuracy under mentally fatiguing conditions, suggesting that the cognitive benefits of stimulation are more pronounced in complex, decision-heavy tasks representative of real-game situations. While subjective fatigue (i.e., the feeling of being mentally “drained”) may not always be alleviated by tDCS, certain facets of performance—in particular, decision-making speed and accuracy—can be preserved or enhanced.

A systematic review with meta-analysis also confirmed that tDCS improves visuomotor skills in athletes. Although meta-regression analyses showed that effect sizes were not significantly related to stimulation parameters, other variables—such as training experience, gender, and genetic predisposition—may modulate the effectiveness of tDCS [78].

### 4.5. Timing, Duration, and Location of Stimulation

The reviewed studies employed a wide range of stimulation parameters, including current intensities from 0.5 mA to 2 mA, durations ranging from 15 to 30 min, and electrode placements targeting the motor cortex, premotor cortex, DLPFC, visual cortex, or bilateral configurations. Findings consistently emphasize that stimulation of the motor cortex—particularly when combined with skill practice—produces the most reliable performance gains, whereas outcomes from other electrode montages are more variable.

Several studies [66,68,74] have reported delayed rather than immediate benefits, highlighting how the effects of tDCS on synaptic plasticity and memory consolidation may take time to fully manifest. These findings suggest that neural facilitation induced by tDCS may bolster subsequent consolidation processes, resulting in performance improvements that become evident only at later testing points.

## 5. Mechanisms of Action of tDCS in Improving Sports Performance

Although the results regarding the effect of tDCS on improving performance in basketball players shown in this review are exploratory, preliminary, and often mixed, tDCS may indeed improve performance in athletes from many sports. It may be valuable to discuss the possible mechanisms of action of tDCS in sports to decipher the physiological effects of stimulation, as well as to assist future researchers in designing future studies in this area and interpreting the results. The mechanisms of action of tDCS have been derived from many sports, which are discussed below.

### 5.1. Cortical Excitability

tDCS can modulate cortical neuron excitability by shifting their resting membrane potential. Anodal tDCS induces a partial depolarization of neurons in the targeted region, making them more likely to fire, whereas cathodal current tends to hyperpolarize and inhibit firing. In motor areas such as the primary motor cortex (M1), anodal stimulation increases cortical excitability and corticospinal output, evidenced by larger motor-evoked potentials and elevated firing rates. These excitability changes can last beyond the stimulation period (e.g., ~60–90 min after a 10–20 min stimulation) due to synaptic plasticity mechanisms (see the Synaptic Plasticity subsection below) [79]. Heightened cortical excitability is a fundamental neurophysiological pathway for performance enhancement because it primes the motor cortex for greater activation during exercise [80]. Notably, the increased excitability of M1 reduces the need for excessive input from higher-order motor regions like the supplementary motor area (SMA) during exercise [81]. The SMA normally contributes to motor drive especially as a task becomes more effortful, but its activity is closely linked to the sensation of effort/fatigue [82,83]. By relying less on the SMA (thanks to a more excitable M1), the brain generates the required motor output with lower perceived effort [84,85]. In this way, anodal tDCS elevates cortical excitability in target regions (e.g., M1, dorsolateral prefrontal cortex) and reorganizes inter-cortical contributions, setting the stage for improved motor performance with reduced subjective effort.

### 5.2. Motor Output and Descending Drive

Enhancing motor cortical excitability translates into greater motor output to the muscles. Anodal tDCS over M1 increases the descending corticospinal drive, effectively “up-regulating” the motor command signal for a given voluntary effort [86,87]. As a result, athletes can recruit motor units more fully or efficiently. Studies have shown, for example, that anodal M1 stimulation can increase maximal force or the time to exhaustion in sustained contractions by boosting output from M1 and delaying the onset of central fatigue [88,89]. In one study, tDCS increased corticospinal excitability of knee extensor representations and was hypothesized to augment output to working muscles with less central effort, correlating with a longer time to exhaustion [90]. Electrophysiologically, tDCS has been observed to alter muscle electromyographic (EMG) activity patterns: some experiments report higher EMG amplitudes or altered motor unit recruitment strategies after stimulation [91,92]. For instance, anodal stimulation of the motor or prefrontal cortex can lead to increased EMG in the prime mover muscles during exercise, suggesting more motor units are activated or maintained [91,92]. Together, these effects mean that for strength-based performance, tDCS may acutely improve peak force and power output by facilitating the neural drive needed to fully activate muscles. For example, a single session of anodal tDCS to DLPFC has been shown to increase training volume and preserve higher movement velocity in resistance exercises [93], likely by sustaining motor unit activation as fatigue would normally set in. In endurance tasks, an elevated descending drive helps delay the point at which the central nervous system can no longer adequately activate the muscles (i.e., postponing central fatigue), thereby prolonging exercise duration [94,95]. Overall, by increasing the vigor of motor commands from brain to muscle, tDCS can acutely enhance muscle recruitment and work output, benefiting both maximal efforts and sustained submaximal exercise.

### 5.3. Fatigue Resistance: Central and Peripheral

Central fatigue—the progressive inability of the central nervous system to drive the muscles [96,97]—is a major limiting factor in endurance performance. tDCS appears to improve resistance to central fatigue through several converging actions. Firstly, by increasing M1 excitability and descending drive (as described above), tDCS delays the onset of supraspinal fatigue; the motor cortex can continue to generate required outputs at a stage when, without stimulation, it might be failing to fully activate motor neurons [98]. Secondly, tDCS may modulate the brain’s fatigue-monitoring networks (sometimes conceptualized as a “central governor”) [99]. This network includes cortical and subcortical regions such as the thalamus, insular cortex, anterior cingulate cortex (ACC) [100], which integrate signals of effort and homeostasis to regulate exercise intensity. Under heavy exercise, increased activity in insulo-cingulate regions and the SMA is associated with the rising sensation of fatigue and the decision to reduce effort [101]. Anodal tDCS can tilt the balance of this network by boosting facilitatory output from M1 and related circuits, thereby counteracting inhibitory fatigue signals. Empirically, many studies have reported an extended time to exhaustion and improved endurance after anodal tDCS, especially in paradigms where central (neural) fatigue is the primary cause of task failure. For example, stimulating M1 allowed participants to cycle longer at a fixed intensity, presumably because their brains could maintain drive despite accumulating central fatigue signals [102]. This aligns with findings that subjects often quit endurance exercise due to central fatigue before peripheral muscles are completely spent. By alleviating central fatigue, tDCS enables athletes to approach their true physiological limits.

Peripheral fatigue refers to the loss of force generation capacity within the muscles themselves (e.g., due to metabolic accumulation, impaired contractile function) [103]. tDCS does not act directly on muscle fibers, but it can still influence peripheral fatigue indirectly. One way is by allowing more even or prolonged motor unit recruitment, which might delay the local accumulation of metabolites in any one set of fibers. Another indirect route is via pacing and distribution of effort: with reduced central fatigue and perceived exertion, athletes might sustain a steadier output rather than lapsing into inefficient force fluctuations, thereby optimizing muscle energy use. There is also evidence that tDCS can improve oxygen delivery or utilization by modulating autonomic functions (discussed below), which could slow the development of peripheral fatigue. However, when an exercise is highly localized and limited mainly by peripheral factors, tDCS shows less effect. In other words, if muscle fibers themselves are the bottleneck, increasing brain drive may not overcome that limit and can even hasten peripheral exhaustion by pushing the muscle harder. In practice, endurance performance of large muscle groups or whole-body exercise, which is usually terminated by central fatigue, benefits more from tDCS than small-muscle isolated tasks. In summary, tDCS chiefly extends fatigue resistance by combating central neural fatigue—keeping the motor “engine” running—and, to a lesser extent, by improving conditions that mitigate peripheral fatigue.

### 5.4. Perceived Exertion (RPE)

Ratings of perceived exertion (RPE)—the subjective sense of effort and fatigue during exercise—are consistently lower after tDCS [104], indicating a powerful psychological pathway for performance gains. The perception of effort is thought to arise from a combination of feedforward motor command signals and feedback from muscles and organs [105]. By altering these signals, tDCS effectively changes how hard exercise feels at a given workload. Anodal stimulation of the left dorsolateral prefrontal cortex (DLPFC) and of insula-adjacent regions (via temporal cortex electrode placement) have each been shown to attenuate RPE. For instance, placing the anode over the temporal cortex (targeting the insular cortex, IC) in cyclists led to reduced RPE during exercise and a higher power output at exhaustion [94]. Similarly, stimulating the DLPFC has lowered perceived exertion in both strength and endurance contexts: one study found enhanced training volume and lower RPE during resistance exercise after DLPFC tDCS [106], and others reported that DLPFC stimulation prolonged cycling time-to-exhaustion with a concomitant drop in RPE compared to sham [102]. These changes in exertional feelings are not merely subjective but have direct performance consequences, since a lower RPE allows athletes to push closer to their physiological limits before mental cutoff. Mechanistically, tDCS-induced RPE reduction is tied to the earlier points about cortical drive and SMA involvement. Because anodal tDCS enables a given output to be achieved with lower central motor command (less corollary discharge signaling effort), the brain registers less effort for the same physical work. In essence, the normal sensory feedback and corollary signals that inform the brain about “how hard I’m working” are modulated. The insular cortex, which integrates visceral fatigue signals and contributes to the feeling of exertion [107], may be hypoactivated by tDCS, thereby damping the intensity of fatigue-related interoceptive signals. The DLPFC, on the other hand, plays a role in the appraisal of effort and in top-down attentional regulation [108]. Anodal DLPFC stimulation likely enhances executive processes that help suppress or reframe fatigue signals, enabling the athlete to tolerate effort for longer [109]. It has been hypothesized that the decision to terminate exercise at volitional exhaustion is fundamentally a cognitive one made in prefrontal circuits [110]. By keeping these circuits engaged and optimistic (for example, through increased motivation or decreased perception of negative bodily signals), tDCS delays the point at which the athlete decides the effort is unbearable. In summary, reduction of perceived exertion is one of the most consistently reported effects of tDCS during exercise and is a key psychological mechanism by which performance is improved. Lower RPE means the athlete perceives the task as easier, which, in turn, allows a higher work rate or longer duration before hitting subjective exhaustion.

### 5.5. Autonomic Nervous System Activity

Beyond the central motor and sensory effects, tDCS can influence the autonomic nervous system (ANS), which controls heart rate, blood pressure, and other visceral functions crucial to exercise performance [111]. Stimulation over the insular cortex (via temporal placements) and the prefrontal cortex has been linked to changes in autonomic outflow. The insula is a critical cortical node in autonomic regulation [112]; left insular stimulation, for example, tends to enhance parasympathetic (vagal) activity, whereas the right insula is more associated with sympathetic drive. In line with this, anodal tDCS targeting the left IC region has produced a lower heart rate during exercise and a delayed withdrawal of vagal tone. Okano et al. observed that cyclists who received left temporal (insula-targeting) tDCS had significantly reduced heart rates at submaximal intensities, and the normal rise in heart rate (due to vagal withdrawal and sympathetic activation) was blunted or delayed relative to sham [94]. In other words, their cardiovascular system was more efficient, staying in a parasympathetically moderated state longer into the exercise. This has obvious benefits: a lower heart rate for a given workload means less cardiac strain and better endurance (since high heart rates correlate with fatigue). Enhanced vagal activity also supports better oxygen delivery and metabolic control [113,114]. Indeed, there is a well-known correlation between aerobic fitness and vagal tone (highly fit individuals show greater heart rate variability and quicker recovery, indicative of strong parasympathetic modulation) [115]. tDCS may acutely shift an athlete’s autonomic state toward that of a “fitter” person, with a calmer cardiovascular response. In addition to heart rate effects, tDCS might influence blood pressure and blood flow regulation through central autonomic networks. DLPFC stimulation, for instance, could reduce sympathetic stress responses by top-down inhibition of limbic centers, thereby keeping blood pressure lower during stressful exercise bouts (though direct evidence on blood pressure is limited). The overall autonomic modulation likely contributes to improved endurance performance [116]: in one case report with a spinal cord injury patient, anodal M1 tDCS extended exercise duration alongside a prolonged time to reach a heart rate variability threshold, implying delayed sympathetic domination of heart rate [98]. The authors attributed the increased exercise tolerance partly to ANS modulation by tDCS. Additionally, ANS changes intertwine with perceived effort and fatigue—a calmer ANS response can make exercise feel less taxing. By modulating cortical areas involved in autonomic control (such as the insula and medial prefrontal cortex), tDCS provides an avenue to optimize the unconscious physiological support for exercise. This mechanism is especially pertinent for endurance exercise, where cardiovascular and thermoregulatory efficiency can govern performance. However, even in strength or high-intensity efforts, a balanced autonomic response (e.g., preventing excessive sympathetic overdrive) can improve focus and reduce premature fatigue. In summary, tDCS can subtly recalibrate autonomic function during exercise—lowering heart rate, sustaining vagal activity, and improving homeostatic control—thereby contributing to an athlete’s ability to sustain effort.

### 5.6. Synaptic Plasticity and Training Adaptation

While many of the above effects occur in the immediate aftermath of stimulation, tDCS also engages mechanisms of synaptic plasticity that can lead to longer-term performance enhancements. Repeated sessions of tDCS or pairing tDCS with training may produce lasting changes in neural circuit connectivity and efficiency. At the cellular level, anodal tDCS induces long-term potentiation-like (LTP-like) effects in stimulated neurons [117]. Experiments in animal models have shown that direct current stimulation can produce sustained increases in synaptic strength that depend on N-methyl-D-aspartate (NMDA) receptor activation and calcium influx—the hallmarks of Hebbian LTP. Notably, tDCS-driven LTP is associated with enhanced secretion of brain-derived neurotrophic factor (BDNF) and activation of its TrkB receptors, which are crucial for activity-dependent synaptic plasticity [118]. If BDNF signaling is blocked or genetically impaired, the facilitatory effects of tDCS on synapses and learning are abolished. This indicates that tDCS taps into the molecular pathways of learning and memory. In human studies, anodal tDCS over M1 has been shown to improve motor skill acquisition and retention [119], consistent with an LTP-like strengthening of motor circuits. For sport performance, such plastic changes could mean faster learning of complex motor skills (technique refinement) and possibly greater long-term gains from training. For example, pairing tDCS with a motor training session can lead to more pronounced improvements in skill or strength than training alone, and these gains can persist as the brain networks have been nudged into a more plastic, learning-ready state. Over time, synaptic modifications in the motor cortex and associated regions might also upregulate the baseline level of corticospinal excitability, essentially “hard-wiring” some of the acute benefits described earlier. In endurance sports, repetitive tDCS could condition brain networks to better resist fatigue or discomfort by reinforcing pathways that inhibit negative signals (akin to cognitive resilience training). Although direct evidence in athletes is still emerging, the principle is that tDCS not only provides an acute boost but also serves as a primer for neuroplasticity. By promoting the growth of stronger synaptic connections in motor and cognitive circuits, tDCS can enhance the effects of practice—whether practicing lifting a heavier weight or sustaining a target pace. This is supported by the understanding that tDCS causes changes in dendritic spine morphology and synaptic protein expression (e.g., increased AMPA receptor phosphorylation and neurotrophin levels) [120], which mirror the effects of intense practice or exercise on the brain. In summary, tDCS engages synaptic plasticity mechanisms (NMDA receptor-dependent potentiation and BDNF-driven synapse strengthening) that may underlie not only the prolonged after-effects (tens of minutes) of a single stimulation but also cumulate with repeated sessions to produce durable improvements in motor performance and learning. This plasticity-focused pathway complements the immediate neurophysiological and psychological effects of tDCS, ultimately contributing to enhanced sport performance both acutely and in the long run.

## 6. Limitations and Future Directions

While the collective findings suggest that tDCS holds promise for enhancing specific aspects of basketball performance, several limitations must be acknowledged.

First, sample sizes across the reviewed studies were generally modest, often comprising fewer than 30 participants per group. Such small sample sizes reduce statistical power and may limit the generalizability of findings to broader populations, including professional athletes, youth players, or female athletes outside the college-age range. Moreover, across the 11 trials, participant samples ranged from junior-high athletes (~16 years, [67]) to professional adults (>24 years, [73]); from novices who were deliberately screened for low skill (e.g., [71]) to elite national-level players ([69,73]); and from exclusively male cohorts to all-female or mixed-sex groups. Given that factors such as age, sex, skill level, and training load influence the rate of motor skill acquisition and consolidation, substantial biological variability is likely embedded within the observed effect sizes. When such differences are unevenly distributed across real and sham tDCS conditions, they introduce unexplained variance and compromise the validity of direct between-group comparisons. Larger-scale trials, potentially involving multi-center collaborations, are needed to strengthen the evidence base and permit more reliable subgroup analyses (e.g., based on skill level, sex or age).

Second, despite multiple investigations targeting basketball-specific performance, there remains considerable heterogeneity in stimulation protocols. These differences include electrode montages, current intensities (ranging from 0.5 mA to 2 mA), electrode sizes and placements, and session durations of 15–30 min. This lack of standardization hinders the identification of optimal tDCS parameters. Moreover, even when similar protocols are employed, outcomes can vary widely, suggesting that individual factors—such as baseline skill level, cortical excitability, and genetic predisposition—may influence responsiveness to tDCS. The studies included in this review, as well as other reviews, suggest that stimulation of the primary motor cortex (M1) and left dorsolateral prefrontal cortex (DLPFC) may be promising cortical targets for improving performance in athletes. Future studies should investigate the effects of tDCS using these montages in future studies. Future research should explore dose–response relationships systematically using standardized tDCS procedures (e.g., consistent electrode placement, current density, and total stimulation time) while controlling for individual differences in brain anatomy, training background, and cortical excitability.

Third, the review aggregated at least 14 distinct outcome domains, including five variants of shooting accuracy, dribbling time, jump height, repeated-sprint speed, reaction time to deceptive cues, several indices of subjective and physiological fatigue, visuomotor precision, decision-making under fatigue, visual-search metrics, and inhibitory control performance. Even within the shooting domain, protocols differed considerably in terms of distance (e.g., free throw, two-point, three-point, five-spot composite), scoring method (binary hit/miss vs. four-point scales), and timing of assessment (immediate, 24 h, 7 days, 21 days). These constructs differ in measurement error and sensitivity to change, resulting in substantial statistical heterogeneity (I^2^). Pooling such outcomes in a single meta-analysis would violate the assumption of a shared underlying effect. To enhance comparability and repeatability, future studies should employ previously validated performance metrics.

Fourth, most current studies have assessed relatively short-term outcomes—typically focusing on improvements in shooting form, decision-making, or sprint performance measured immediately or within a few days or weeks after stimulation. While such outcomes are useful for establishing proof of concept, basketball performance unfolds over full competitive seasons and within dynamic, high-pressure environments. The translation from controlled lab or training settings to in-game performance remains understudied. Longitudinal research tracking players’ real-world performance metrics—such as shooting percentage, turnover rates, and fatigue profiles—over months or an entire season would help clarify the practical utility of tDCS interventions.

Fifth, some studies have reported delayed benefits that emerged hours or days after stimulation rather than during or immediately following the interventions. Although this temporal pattern is consistent with the neuroplastic changes believed to be promoted by tDCS, the underlying mechanisms remain insufficiently understood. Future studies utilizing neuroimaging techniques (e.g., functional MRI or EEG) or neurophysiological assessment (e.g., transcranial magnetic stimulation to assess motor cortical excitability) could provide valuable insights into the timing, localization, and nature of tDCS-induced plasticity. Such insights could inform the strategic scheduling of tDCS sessions to align with optimal windows for practice, rest, and memory consolidation.

Sixth, while some studies have examined tDCS under conditions of mental fatigue or cognitive overload (e.g., extended video gameplay or prolonged Stroop tasks), the results are inconclusive regarding whether tDCS meaningfully attenuates subjective fatigue. On one hand, some findings suggest that mental fatigue and ratings of perceived exertion can be lowered or that performance decrements under fatigue can be delayed. On the other hand, several studies have reported no significant differences in subjective fatigue levels between active and sham conditions. This inconsistency may stem from differences in the nature of fatigue-induction protocols, as well as the distinction between subjective perceptions and objective performance measures More systematic research is needed to determine the specific fatigue states or cognitive demands under which tDCS exerts the greatest beneficial effect. Identifying these contexts would enhance the practical application of tDCS in both training and competitive environments.

Seventh, the effectiveness of blinding is not always thoroughly reported, and participant expectancy can significantly influence results in performance-based research. Robust blinding (e.g., using low-level sham currents or ramp-up/down techniques) is essential to minimize placebo effects. Furthermore, many studies relied on convenience samples of collegiate or semiprofessional players, which limits the generalizability of the findings to elite athletes performing under higher levels of physical and psychological pressure. Investigating how tDCS interacts with other performance-enhancement strategies (e.g., physical training, mental imagery, observational learning, or nutritional interventions) would also provide valuable insights into its potential role within comprehensive training regimens.

Eighth, sham procedures were inconsistent, ranging from true zero-current conditions to brief ramp-up/ramp-down protocols. Moreover, several studies paired both real and sham stimulation with additional learning interventions (model observation, mental imagery, point-light demonstrations, video games, and music-based distraction). These co-interventions can produce independent training effects, potentially confounding the isolated contribution of tDCS and unpredictably inflating or diminishing between-group contrasts. Future research should isolate the effects of tDCS from the effects of tDCS combined with training.

Ninth, study designs included parallel-group RCTs, crossover RCTs, and mixed single-blind or double-blind formats. Sample sizes ranged from *n* = 8 to *n* = 52, and analytical strategies varied from simple paired *t*-tests to three-way mixed-model ANOVAs. Small, single-center samples are especially prone to imprecise effect estimates; crossover designs introduce potential period and carryover effects; and diverse statistical approaches generate effect sizes that are not directly comparable without re-calculation. These factors further limited the possibility of generating a pooled effect estimate or of applying formal heterogeneity statistics across the corpus. In addition, several studies failed to report complete numerical for key outcomes. These methodological deficiencies preclude valid between-study comparisons and prohibit meta-analyses. Future studies should report full numerical data with results, use robust statistical analyses, and calculate standardized effect sizes. These steps are essential for accurately evaluating the impact of tDCS on athletic performance and for enabling systematic reviews and meta-analyses that can definitively assess its efficacy.

Future research on tDCS in basketball should begin with larger, multisite trials to address the limitation of small sample sizes and heterogeneous participant characteristics. Standardizing stimulation protocols—particularly with respect to electrode placement, current intensity, and session duration—would help identify the most effective parameters for specific performance goals, such as shooting accuracy or sprint endurance. Studies should also extend beyond short-term outcomes to include longitudinal follow-ups and in-game assessments, exploring how tDCS impacts performance under real competitive conditions across full seasons.

Integrating neuroimaging or neurophysiological measures would illuminate the mechanisms by which tDCS modulates cortical networks involved in motor control, skill acquisition, and decision-making. Additionally, research should investigate the potential synergy between tDCS and other training modalities—such as observational learning, mental imagery, and targeted physical conditioning—to determine whether these combinations produce greater improvements than tDCS or conventional training alone.

## 7. Practical Implications

For coaches, athletes, and sports practitioners, this review offers cautious yet intriguing insights into how tDCS may be used as a performance-enhancing tool in basketball and potentially in other team sports.

### 7.1. Skill Training Enhancement

The findings suggest that applying tDCS (particularly anodal stimulation over the motor cortex) during practice sessions may lead to modest improvements in motor skills such as shooting accuracy and dribbling speed. In practical terms, a basketball coach might consider integrating tDCS into training routines for skills that require fine motor control and consistency, including free throws, jump shots, and ball-handling drills.

Given that many studies have reported more substantial improvements at delayed rather than immediately post-intervention, one practical implication is that tDCS could be administered before or during training sessions with the expectation that its benefits will emerge in subsequent performances (e.g., in games the following day or week). Coaches might use this as a tool to enhance learning consolidation, thereby increasing the effectiveness of practice.

### 7.2. Cognitive and Decision-Making Benefits

For cognitive aspects such as decision-making, focus, and resistance to distractions of deceptive maneuvers (e.g., head fakes), the review provides evidence that stimulating frontal brain regions—particularly the left DLPFC—can improve basketball-specific cognitive performance. Practically, this means that teams dealing with issues related to concentration or decision-making under pressure may benefit from incorporating tDCS into their mental training programs.

For example, a point guard might undergo a short tDCS session before engaging in video analysis or situational drills to potentially enhance executive functioning and reduce the chance of falling for opponents’ feints, as suggested in the study on “head-fake” susceptibility. While this application remains speculative, it points to a potential use of tDCS beyond physical training: it could serve as a tool for mental conditioning, helping athletes maintain cognitive sharpness during high-stress, fatigue-inducing periods of competition.

Sports psychologists or performance staff may find tDCS to be a useful adjunct in sustaining high-level cognitive functioning, especially in scenarios where mental fatigue typically impairs performance, such as the fourth quarter of a close game. As the review noted, some studies have found that players maintained decision-making accuracy under conditions of mental fatigue when supported by tDCS. This suggests potential utility in prolonging peak mental performance during critical moments of competition.

### 7.3. Fatigue Management

Fatigue—both physical and mental—is a major factor affecting performance in competitive basketball. The review’s findings on fatigue are mixed and do not clearly suggest that tDCS may help delay performance deterioration due to fatigue, even if subjective fatigue remains unchanged. However, as mentioned earlier, a single stimulation session may not be sufficient to improve this parameter. Fatigue is a complex neurophysiological construct, and it is plausible that only repeated tDCS sessions can elicit meaningful effects. A previously cited review showed that tDCS has the potential to reduce perceived fatigue levels [77].

In practical terms, if tDCS can modestly extend the time before a player’s skills decline from exhaustion, it could prove valuable in a competitive setting. A strength and conditioning coach might incorporate tDCS into recovery or pre-game routines to help players sustain endurance or cognitive focus. For example, before a series of intense scrimmages, applying tDCS to the motor or frontal cortex may allow players to maintain sprint speed and accuracy for a longer period.

The review also notes reduced ratings of perceived exertion in some cases, although this effect was not consistently observed. If future research confirms that tDCS can reliably lower RPE—as suggested in studies beyond those included in this review—athletes might be able to push themselves harder in training or competition due to experiencing less subjective fatigue. The practical implication is that tDCS could become part of a broader fatigue-mitigation strategy, complementing established approaches such as nutrition, hydration, and rest to help athletes maintain peak performance.

### 7.4. Accessibility and Ease of Use

tDCS is noninvasive and relatively easy to administer. Unlike some high-tech interventions that require costly equipment or laboratory settings, tDCS devices are portable and increasingly affordable. This means a team could realistically maintain a tDCS kit in its training facility without incurring significant costs.

Because tDCS is safe and typically produces only a mild tingling sensation, athlete compliance is likely to be high. Players are generally open to trying interventions that may enhance performance if there are no significant risks involved. The review’s synthesis of multiple studies provides reassurance that no major adverse events were reported, and no injuries or harmful side effects were noted when standard protocols were followed. This “low risk, moderate reward” profile positions tDCS as a feasible tool in the coach’s toolbox. For example, integrating tDCS into video sessions or pre-practice warm-ups could eventually become as routine as foam-rolling or icing—assuming continued support from emerging evidence.

### 7.5. Guidance for Implementation

That said, the review also implicitly offers cautionary guidance. Given the variability in outcomes, practitioners should not expect dramatic or universal effects. A key takeaway is the importance of standardized protocols. If a sports scientist or trainer wishes to implement tDCS, they should closely replicate the setups shown to be effective (for instance, anodal stimulation of M1 at 1–2 mA for ~20 min during practice drills) rather than experimenting with untested configurations.

Practitioners should also manage expectations: improvements may be subtle and may not manifest immediately. The review suggests that tDCS is most effective when used in synergy with traditional training methods. Therefore, it should be viewed not as a replacement for practice but as a potential amplifier of training effects. This implies that tDCS should be layered onto existing routines—such as shooting drills, scrimmages, or tactical film sessions—rather than treated as a standalone intervention.

Another practical consideration is timing. Since many observed benefits appeared at delayed follow-up points, coaches should not be discouraged if immediate post-tDCS performance does not show clear improvement. Tracking performance across subsequent days may reveal delayed gains. Although the review emphasizes this for research, coaches could informally monitor athletes’ performance over time to assess any impact.

### 7.6. Beyond Basketball

While this review focuses on basketball, the practical implications likely extend to other sports with similar demands. Sports such as soccer, rugby, or volleyball, which involve high-intensity skill execution under fatigue and rapid decision-making, may also benefit from tDCS interventions. For example, a soccer coach, could consider applying motor cortex tDCS during penalty-kick training (analogous to free-throw shooting) or use DLPFC tDCS for players requiring improved decision-making in the late stages of a match.

The review provides a foundational reference for practitioners in these sports, identifying configurations and methods that have shown efficacy in basketball. However, it also warns them that current evidence is preliminary; tDCS should be treated as an experimental supplement, rather than a guaranteed performance booster.

Importantly, sports governing bodies do not currently ban tDCS use, as it is non-invasive and not pharmacological. While the concept of “neurodoping” has been discussed in the academic literature, no regulations currently exist to restrict its use. This opens the door for early adopters seeking competitive advantages, but it also increases the need for ethical and scientifically grounded implementation.

The practical takeaway from this review is clear: tDCS shows promise, but it should be implemented with caution. Teams should establish proper sham controls when piloting its use internally to rule out placebo effects, monitor each athlete’s individual response, and remain informed as research continues to refine protocols and uncover new insights.

### 7.7. Translating Laboratory Results into Competitive Performance—Is There a Solution?

Research on the application of tDCS in sports typically relies on laboratory-based protocols. Athletic performance is most often measured under controlled conditions, followed by brain stimulation, and then reassessed using psychophysiological performance measures in the same experimental setting. However, such laboratory conditions differ significantly from those encountered during real-world sports competitions. In competitive environments, athletes are exposed to a wide array of uncontrolled variables—including ambient distractions (e.g., noise and lighting), psychological stress, emotional arousal, interpersonal interactions, and situational pressure—that are difficult to replicate in experimental settings.

Ideally, the effects of tDCS should be assessed in real-time during competition to determine its practical utility. However, implementing neurostimulation protocols in competitive contexts poses significant logistical and ethical challenges. While it is more feasible to evaluate the effect of stimulation on performance during regular training sessions, such assessment may not capture the full spectrum of demands encountered in actual competitions—whether brief, precision-based tasks (e.g., archery) or dynamic, multidimensional scenarios typical of team sports like basketball.

A partial solution involves comparing an athlete’s baseline performance during competition—measured across various objective indicators without tDCS—with their performance in subsequent competitions following stimulation. Yet this approach also has limitations. Performance fluctuations across competitions may arise from differences in the athlete’s physiological state, motivation, fatigue level, or recovery status, as well as variability in the difficulty and context of the competitive event itself.

Accordingly, future research must address the challenge of approximating real-world performance conditions as closely as possible, whether through advanced simulation, hybrid field–laboratory designs, or performance monitoring in ecologically valid training environments. Only under such conditions can the field conclusively determine whether tDCS is a viable tool for enhancing athletic performance in competitive settings.

## 8. “Neurodoping” via tDCS—Is There Any Risk?

Physical fatigue is a natural consequence of exertion. As performance deteriorates, muscle strength and endurance decrease—mechanisms regulated by the physiology of the entire body, including the brain. These limitations serve a protective function: they signal the need for rest and prevent disruption of homeostasis, which could otherwise lead to adverse outcomes such as injury. If tDCS “tricks” the brain into overriding these natural physiological boundaries, does its regular use in sports pose a risk of overtraining or injury?

To date, no studies have directly addressed this question, as the application of tDCS in athletic contexts remains in an experimental phase. Nonetheless, this dilemma warrants considerations both in future research and in practical implementations across different sports. If tDCS raises the threshold for perceived fatigue and effort—signals that typically prompt athletes to rest—could this increase the likelihood of exceeding safe limits and lead to harm?

Medicine and sports science must seek answers to these critical questions. Understanding whether tDCS disrupts protective physiological feedback mechanisms is essential before its widespread adoption in elite performance environments can be ethically and safely justified.

To rigorously evaluate the efficacy of tDCS as a form of “neurodoping” in athletic contexts, future research should move beyond self-report measures and basic mechanical performance metrics by incorporating biological markers of physiological response. This approach would provide a more comprehensive understanding of the underlying mechanisms of action and help rule out placebo-driven effects. Biomarkers such as lactate, urea, myoglobin, and creatine kinase (CK)—already widely used in elite sport—can serve as objective indicators of acute internal load and can inform assessments of fatigue, stress, tissue damage, and recovery processes [121]. Additionally, integrating neuroimaging techniques commonly employed in tDCS research, including electroencephalography (EEG) and functional magnetic resonance imaging (fMRI), would allow for the direct observation of neurophysiological effects associated with stimulation. These tools can help elucidate the neural correlates of performance improvements and clarify how tDCS modulates brain function in trained athletes.

## 9. Conclusions

Basketball demands a combination of high-level physical skills, sophisticated decision-making, and resilience under mental and physiological fatigue. Growing evidence suggests that tDCS may beneficially modulate various performance dimensions, including shooting accuracy, dribbling speed, reactive agility, and decision-making. However, the scope of existing findings remains limited by relatively small sample sizes, heterogeneous study designs, and inconsistent stimulation parameters.

Despite these constraints, the reviewed studies collectively point to a positive influence of tDCS on motor learning and skill consolidation. The technique’s noninvasive nature and relative ease of application make it an attractive adjunct to traditional training methods. In particular, targeting the primary motor cortex frequently correlates with improvements in basketball-specific tasks, especially those requiring refined motor coordination, such as free-throw shooting or dribbling maneuvers. Moreover, when tDCS is combined with strategies such as observational learning or mental imagery, longer-term gains in skill retention often emerge, sometimes more clearly observed at follow-up assessments than in immediate post-test measures.

The practical implications are potentially broad, ranging from helping players refine shooting mechanics to maintaining higher decision-making accuracy under mental fatigue. Even so, real-world implementation requires further standardization, with future studies focusing on robust experimental designs that capture in-game performance and longer-term effects. Overall, while tDCS represents a promising intervention within sports science, especially for basketball, additional research is essential to establish clear guidelines on optimal dosing, electrode placement, timing, and synergy with other training modalities. Through concerted efforts in these areas, tDCS may become an increasingly reliable and effective tool for enhancing athletic performance in basketball and beyond.

## Figures and Tables

**Table 1:** The summary of studies included in the review.

Key Findings	Task Type	tDCS Parameters	tDCS Target Area	Participants	Study
Shooting and dribbling improved significantly under tDCS vs. sham.	100-shot accuracy & Illinois ball-dribbling test	1 mA for 20 min	Primary motor cortex (C3 or C4)	52 sports students (19 men, 33 women)	[65]
Anodal tDCS reduced reaction times in deception-based tasks.	Reaction time in head-fake deception task	0.5 mA for 19 min	Left DLPFC (F3)	50 right-handed adults (limited basketball experience)	[66]
Free-throw accuracy improved with tDCS + point-light modeling, with sustained effects.	Free-throw shooting after point-light modeling	1.5 mA for 20 min	Left premotor cortex	26 skilled male basketball players (mean age 16.5)	[67]
No immediate effects, but significant improvements in two-point field throws at post-test and follow-up.	Two-point field throws after point-light modeling	1.5 mA for 20 min	Left premotor cortex	26 skilled male basketball players	[68]
No significant improvement in shooting performance or cognitive workload.	Three-point shooting	2 mA, 20 min	Left and right DLPFC (F3, F4)	8 professional female basketball players	[69]
tDCS increased jump height, sprint speed, and fatigue resistance.	Jump height & repeated sprint performance	2 mA for 20 min	Motor cortex (Cz, C5, C6)	13 trained male basketball players (mean age 20)	[70]
Motor cortex tDCS improved free-throw accuracy more than visual cortex tDCS.	Free-throw shooting (motor vs. visual tDCS)	1.5 mA for 15 min	Motor cortex (C3) & visual cortex (Oz)	45 female university students (novice players)	[71]
tDCS improved three-point shooting under mental fatigue but had no effect on fatigue itself.	Three-point shooting under mental fatigue	1.5 mA for 25 min	DLPFC (F3)	18 male basketball players	[72]
tDCS protected against mental fatigue effects, improving decision-making and visual search efficiency.	Decision-making, visual search, and fatigue assessment	2 mA for 30 min	Motion-sensitive middle temporal area (CP5)	20 professional male basketball players (aged 18–31)	[73]
tDCS with model observation significantly improved free-throw performance, with long-term retention effects.	Free-throw shooting with observational learning	1.5 mA for 15 min	Motor cortex (C3 anode, Fp2 cathode)	30 novice female basketball players	[74]
tDCS led to greater improvements in free-throw accuracy compared to mental imagery, with sustained skill retention over time	Free-throw shooting with mental im.agery vs. tDCS	2 mA for 20 min	Motor cortex (M1, left hemisphere)	36 non-elite basketball players (18–25 years old)	[75]

## Data Availability

No new data were created or analyzed in this study. Data sharing is not applicable to this article.
